# Self-citation policies and journal self-citation rate among Critical Care Medicine journals

**DOI:** 10.1186/s40560-021-00530-2

**Published:** 2021-01-26

**Authors:** Filippo Sanfilippo, Stefano Tigano, Alberto Morgana, Paolo Murabito, Marinella Astuto

**Affiliations:** 1 grid.412844.f Department of Anaesthesia and Intensive Care, A.O.U., “Policlinico-Vittorio Emanuele” University Hospital, via S. Sofia 78, 95100 Catania, Italy; 2 grid.8158.4 0000 0004 1757 1969 .School of Anaesthesia and Intensive Care, University Hospital “G. Rodolico”, University of Catania, Catania, Italy; 3 grid.411489.1 0000 0001 2168 2547 School of Anaesthesia and Intensive Care, Department of Medical and Surgical Sciences, “Magna Graecia” University, Catanzaro, Italy

**Keywords:** Intensive Care, Authors, Citations, Impact factor, Journals, Policies, Self-citations

## Abstract

**Background:**

Inappropriate authors’ self-citation (A-SC) is a growing mal-practice possibly boosted by the raising importance given to author’s metrics. Similarly, also excessive journals’ self-citation (J-SC) practice may factitiously influence journal’s metrics (impact factor, IF). Evaluating the appropriateness of each self-citation remains challenging.

**Main body:**

We evaluated the presence of policies discouraging A-SC in Critical Care Medicine (CCM) journals with IF. We also calculated the J-SC rate of these journals. In order to evaluate if J-SC rates are influenced by the focus of interest of CCM journals, we separated them in three sub-categories (“multidisciplinary”, “broad” or “topic-specific” CCM journals).

We analyzed 35 CCM journals and only 5 (14.3%) discouraged excessive and inappropriate A-SC. The median IF was higher in CCM journals with A-SC policies [4.1 (3–12)] as compared to those without [2.5 (2–3.5); *p* = 0.02]. The J-SC rate was highly variable (0–35.4%), and not influenced by the presence of A-SC policies (*p* = 0.32). However, J-SC rate was different according to the focus of interest (*p* = 0.01): in particular, it was higher in “topic-specific” CCM journals [15.3 (8.8–23.3%)], followed by “broad” CCM [11.8 (4.8–17.9%)] and “multidisciplinary” journals [6.1 (3.6–9.1%)].

**Conclusions:**

A limited number of CCM journals have policies for limiting A-SC, and these have higher IF. The J-SC rate among CCM journals is highly variable and higher in “topic-specific” interest CCM journals. Excluding self-referencing practice from scientific metrics calculation could be valuable to tackle this scientific malpractice.

## Background

Inappropriate authors’ self-promoting is a growing mal-practice [1], possibly prompted by the mounting scientific importance of author’s metrics. We recently surveyed the submission guidelines of Anaesthesiology journals for the presence of policies discouraging author self-citations (A-SC) [2]*.* In parallel, we also evaluated the journals’ self-citation (J-SC) practice, which may hinder attempts of the Editorial Board to increase the journal impact factor (IF).

Although excessive and inappropriate A-SC and J-SC are two independent forms of suboptimal academic practice that have not yet received enough attention, it must be clear from the beginning that not all A-SC and J-SC are synonymous of malpractice.

In the present study, we evaluated the J-SC rate among the Critical Care Medicine (CCM) journals and possible factors influencing it, as well as the presence of policies regarding A-SC in these journals.

## Methods

On the 30 October 2020, we evaluated the presence of policies discouraging A-SC in the submission guidelines of CCM journals selected from the *InCites Journal Citation Reports 2019* (Clarivate Analytics®) [3]. The J-SC rate was evaluated according to the following formula:

$$ \mathrm{JSC}\ \mathrm{rate}=\frac{\mathrm{IF}-\mathrm{IF}\ \mathrm{without}\ \mathrm{self}\ \mathrm{citations}}{\mathrm{IF}} $$

Our previous investigation suggested that journals focusing on a specific topic of the discipline had a trend towards higher J-SC rate (25% vs 7% as compared to those with broad interest; *p* = 0.06) [2]. Accordingly, we performed a secondary analysis separating CCM journals in three sub-categories: (1) “multidisciplinary journals” (defined as journals focusing not only on CCM but also on other disciplines as Anaesthesiology, Respiratory Medicine, etc.); (2) “broad interest” CCM journals (defined as those publishing on several aspects of the discipline); (3) “topic-specific” CCM journals (focusing on specific topics of CCM interest). We evaluated also if J-SC is influenced by presence of A-SC policies.

Continuous variables are presented as median (25th–75th percentile), categorical variables as numbers/percentage. Mann-Whitney *U* test or Kruskal-Wallis test for unrelated samples were performed according to the number of groups. Tests were two-sided; *p* < 0.05 was considered statistically significant.

## Results

A total of 36 CCM journals were found but we removed *Human Gene Therapy Clinical Development* as its scope clearly falls outside the CCM. Therefore, 35 journals were included. In Table 1, we report J-SC rates and presence of policies regarding A-SC. Five journals (14.3%) discouraged “excessive and inappropriate” self-citations; none proposed an A-SC cut-off. The median IF of CCM journals was 2.7 (2.1–3.8) and was higher in CCM journals with policies on A-SC[4.1 (3–12)] as compared to those without [2.5 (2–3.5); *p* = 0.02].

**Table 1:** List of Critical Care Medicine journals according to their rank in Journal Citation Report 2019. For each journal we provide the following: Journal Rank and Full Title, Impact Factor (IF) and IF without self-citation (SC), Journal Self-Citation Rate (J-SC, year 2019), Publisher name, presence and description of policies on limiting SC. Each Journal’s name contains a hyperlink to its “instruction to authors/submission guidelines” so that readers may check them

Journal rank and full title		IF	IF without SC	J-SC rate (2019)	Publisher	Policy description
**1**	Lancet Respiratory Medicine	25,09	24,31	3,1	Elsevier SCI LTD, Oxford, England,	*None*
**2**	Intensive Care Medicine	17,68	15,79	10,7	Springer, New York, USA	Research articles and non-research articles (e.g. opinion, review, and commentary articles) must cite appropriate and relevant literature in support of the claims made. Excessive and inappropriate self-citation or coordinated efforts among several authors to collectively self-cite is strongly discouraged.
**3**	American Journal of Respiratory and Critical Care Medicine	17,45	15,95	8,6	Amer Thoracic Soc, New York, USA	*None*
**4**	Chest	8,31	7,91	4,8	Amer Coll Chest Physicians, Northbrook, USA	*None*
**5**	Critical Care Medicine	7,41	6,78	8,6	Lippincott Williams & Wilkins, Philadelphia, USA	*None*
**6**	Critical Care	6,41	5,89	8,1	BioMed Central LTD, London, England	Research articles and non-research articles (e.g. opinion, review, and commentary articles) must cite appropriate and relevant literature in support of the claims made. Excessive and inappropriate self-citation or coordinated efforts among several authors to collectively self-cite is strongly discouraged.
**7**	Resuscitation	4,21	2,85	32,4	Elsevier Ireland Ltd., Elsevier House, Clare, Ireland	*None*
**8**	Annals of Intensive Care	4,12	3,90	5,5	Springer, New York, USA	Research articles and non-research articles (e.g. Opinion, Review, and Commentary articles) must cite appropriate and relevant literature in support of the claims made. Excessive and inappropriate self-citation or coordinated efforts among several authors to collectively self-cite is strongly discouraged.
**9**	Critical Care Clinics	3,80	3,80	0	W B Saunders Co-Elsevier Inc., Philadelphia, USA	*None*
**10**	Journal of Neurotrauma	3,79	3,46	8,8	Mary Ann Liebert Inc., New Rochelle, USA	*None*
**11**	Journal of Trauma and Acute Care Surgery	3,38	2,77	17,9	John Ewers Wolters Kluwer Baltimore, USA	*None*
**12**	Journal of Intensive Care Medicine	3,14	3,12	0,6	Sage Publications Inc, Thousand Oaks, USA	*None*
**13**	Journal of Intensive Care	3,10	2.96	4,7	BMC, Crinan St., London, England	Research articles and non-research articles (e.g. Opinion, Review, and Commentary articles) must cite appropriate and relevant literature in support of the claims made. Excessive and inappropriate self-citation or coordinated efforts among several authors to collectively self-cite is strongly discouraged.Authors should not preferentially cite their own or their friends’, peers’, or institution’s publications.
**14**	Shock	2,96	2,71	8,3	Lippincott Williams & Wilkins, Philadelphia, USA	Research articles and non-research articles (e.g. Opinion, Review and Commentary articles) must cite appropriate and relevant literature in support of the claims made. Excessive and inappropriate self-citation or coordinate efforts among several authors to collectively self-cite is strongly discouraged
**15**	Current Opinion in Critical Care	2,92	2,81	3,8	Lippincott Williams & Wilkins, Philadelphia, USA	*None*
**16**	Pediatric Critical Care Medicine	2,85	2,26	21	Lippincott Williams & Wilkins, Philadelphia, USA	*None*
**17**	Neurocritical Care	2,72	2,45	10	Humana Press Inc., Totowa, USA	*None*
**18**	Anaesthesia Critical Care & Pain Medicine	2,71	2,32	14,1	Elsevier France-Editions Scientifiques Medicales Elsevier, Issy-Les-Moulineaux, France	*None*
**19**	Journal of Critical Care	2,68	2,55	5,1	W B Saunders CO-Elsevier Inc., Philadelphia, USA,	*None*
**20**	Minerva Anestesiologica	2,50	1,61	35,4	Edizioni Minerva Medica, Turin, Italy	*None*
**21**	Critical Care and Resuscitation	2,49	2,32	6,9	Australasian Med Publ Co Ltd., Australia	*None*
**22**	Medicina Intensiva	2,36	1,71	27,8	Elsevier Espana Slu, Barcelona, Spain	*None*
**23**	Australian Critical Care	2,21	1,89	14,5	Elsevier Science Inc., New York, USA	*None*
**24**	Injury-International Journal of the Care of the Injured	2,11	1,79	14,9	Elsevier Sci Ltd., The Boulevard, Oxford, England	*None*
**25**	American Journal of Critical Care	2,10	1,96	6,8	Amer Assoc Critical Care Nurses, Aliso Viejo, USA	*None*
**26**	Burns	2,07	1,62	21,5	Elsevier Sci Ltd., The Boulevard, Oxford, England	*None*
**27**	Respiratory Care	2,07	1,71	17,4	Daedalus Enterprises Inc., Irving, USA	*None*
**28**	Seminars in Respiratory and Critical Care Medicine	2,03	1,97	2,8	Thieme Medical Publ Inc., New York, USA	*None*
**29**	Anaesthesia and Intensive Care	1,54	1,36	11,8	Australian SOC Anaesthetists, Edgecliff, Australia	*None*
**30**	Journal of Burn Care & Research	1,53	1,29	15,7	Lippincott Williams & Wilkins, Philadelphia, USA	*None*
**31**	Critical Care Nurse	1,48	1,44	2,9	Amer Assoc Critical Care Nurses, Aliso Viejo, USA	*None*
**32**	Therapeutic Hypothermia and Temperature Management	1,18	0,84	28,7	Mary Ann Liebert, Inc., New Rochelle, USA, NY	*None*
**33**	Journal of Trauma Nursing	0,87	0,80	8,8	Lippincott Williams & Wilkins, Philadelphia, USA	*None*
**34**	Anasthesiologie & Intensivmedizin	0,84	0,58	30,4	Aktiv Druck & Verlag GMBH, Ebelsbach, Germany	*None*
**35**	Anasthesiologie Intensivmedizin Notfallmedizin Schmerztherapie	0,53	0,50	5,1	Georg Thieme Verlag KG, Stuttgart, Germany	*None*

The J-SC rate was highly variable (0–35.4%) with median 8.8% (5.1–17.4%) and was not different between journals with or without A-SC policies [8.1% (5.1–9.5%) vs 9.4% (5–18.7%), respectively; *p* = 0.32].

CCM journals were sub-categorized as follows: 11 were considered “multidisciplinary” (Table 1: rank 1-3-4-11-18-20-27-28-29-34-35), 14 were classified as “broad interest” (rank 2-5-6-8-9-12-13-15-19-21-22-23-25-31), and 10 were “topic-specific” CCM journals (rank 7-10-14-16-17-24-26-30-32-33). We found significantly different J-SC rate according to the journal interest (*p* = 0.01), with higher values in “topic-specific” [15.3 (8.8–23.3%)] followed by “broad interest” [11.8 (4.8–17.9%)] and “multidisciplinary” journals [6.1 (3.6–9.1%)]. Conversely, the IF was not different between journals according to sub-categories (*p* = 0.35): “topic-specific” [2.4 (1.4–3.2)], “broad interest” [2.5 (1.5–8.3)] and “multidisciplinary” journals [3.0 (2.3–4.7)]. The J-SC rate was not significantly influenced by the presence of policies on A-SC (8.1% [5.1–9.5%]) or not (9.4% [5–18.7%]; *p* = 0.32).

## Discussion

Our investigations follows a similar assessment conducted in Anaesthesiology journals [2] and found lower prevalence of A-SC policies among CCM journals as compared to Anaesthesiology ones (14% vs 22%). This finding reinforces our belief that the argument of limiting the malpractice of inappropriate self-referencing is still at embryonic level. Several forms of research misconduct (fabrication, falsification, plagiarism, ghost-writing) have been recognized [4, 5], but “inappropriate self-citation” and “citation farms” have not yet received widespread editorial attention. In our opinion, it is urgent to embrace a debate on the best approach for limiting inappropriate self-referencing, keeping in mind that it remains challenging to define the appropriateness of each A-SC [6] and that a single cut-off is unlikely to fit all the manuscript types. Interestingly, we found a significantly higher IF in CCM journal with policies on A-SC, and this may be a marker of higher publishing standards. Such finding is new, as we did not report differences in IF among Anaesthesiology journals according to presence of A-SC policies [2]. This result may support larger investigations, as the findings of single disciplines are likely influenced by a reduced sample size investigated.

The J-SC rate and its variability were similar between CCM (8.8%, range 0–35.4%) and Anaesthesiology journals (8.4%, range 1.4–37.2%), and J-SC rate was not different according to the presence of A-SC policies.

Several factors may influence J-SC rate. Although it is possible that an excessive J-SC rate may sometimes hinder a sort of editorial malpractice (requests to reference specific articles with the aim to boost IF) [7], this is difficult to assess. Conversely, it must be noted that J-SC rate is also increased by editorials and commentaries introducing the highlights of important articles published by the journal. Therefore, the interpretation of J-SC rate is another challenging aspect of self-promoting. Importantly, whilst the presence of A-SC policies does not seem to influence the J-SC rate, our secondary analysis showed that the focus of interest of CCM journals is a factor significantly influencing the J-SC rate. Indeed, CCM journals with narrower focus of interest had significantly higher J-SC as compared with “broad CCM interest” and “multidisciplinary” journals. Therefore, while a narrower focus of interest should not be regarded as justification for excessive self-promoting, the interpretation of J-SC rates should be paired, among others, with critical estimation of the journal’s scope. Conversely, we found that the focus of interest does not seem to influence the journal IFs.

Considering all the factors influencing the J-SC rates and also the difficulties in critically evaluating the appropriateness of each A-SC or J-SC, an option could be to calculate author’s and journal’s scientific metrics excluding self-citations. Doing so, inappropriate self-referencing will become a useless practice. Importantly, Scopus® and Web of Science® databases offer the opportunity to exclude A-SC when observing scientific metrics. Similarly, it is feasible to calculate the journal’s IF without J-SC contribution (as shown in Table 1 in our study).

### Limitations

Overall, our investigation on A-SC policies and J-SC rates over-simplifies complex issues, since we again reinforce that it is challenging to evaluate the appropriateness of each A-SC as well as to show editorial requests to add specific citations. Furthermore, the lack of policies regarding A-SC and the J-SC rate may be greater in “predatory journals” [8, 9]. Another limitation of this study is that several journals publishing in the field of CCM were not included as not listed in the *InCites Journal Citation Reports.* For instance, several Anaesthesiology journals have dedicated sections on CCM.

## Conclusions

We found a very limited number of CCM journals with policies for limiting A-SC. Journals with A-SC policies had higher IF. The J-SC rate was highly variable and greater in journals with narrower focus of interest. Excluding author’s and journal’s self-referencing from the scientific metrics calculation could be a valuable option to tackle these forms of scientific malpractice.

## Data Availability

Available on request and online at the source quoted.

## Abbreviations

A-SC: Author’s self-citation
CCM: Critical care medicine
IF: Impact factor
J-SC: Journals’ self-citation
